# Craniospinal irradiation in the treatment of chemotherapy refractory leptomeningeal metastasis from breast cancer: A case report

**DOI:** 10.1002/cnr2.1556

**Published:** 2021-11-10

**Authors:** Daniel Tesolin, Dimitrios Vergidis, Kevin Ramchandar

**Affiliations:** ^1^ Northern Ontario School of Medicine Thunder Bay Ontario Canada; ^2^ Providence Health Hematology/Oncology St. Paul's Hospital Vancouver British Columbia Canada; ^3^ Regional Cancer Care Northwest Thunder Bay Regional Health Sciences Centre Thunder Bay Ontario Canada

**Keywords:** breast cancer, craniospinal irradiation, leptomeningeal metastasis

## Abstract

**Background:**

Leptomeningeal carcinomatosis is a relatively uncommon complication of solid tumors that is associated with significant morbidity and mortality. Prognosis is typically weeks to months and the neurologic complications of this disease can significantly affect quality of life. The role of craniospinal irradiation is unclear as evidence exploring this treatment option is limited. Despite lack of evidence, its use has decreased due to its associated acute toxicities and newer intrathecal alternatives.

**Case:**

Here we report the case of a 50‐year‐old patient who received craniospinal irradiation for chemotherapy‐refractory leptomeningeal disease, with survival well beyond the median and good quality of life for the majority of that time.

**Conclusion:**

This patient's remarkable survival and performance after treatment suggests that craniospinal irradiation could be considered more frequently in the treatment of leptomeningeal metastases. To our knowledge, this is the first case with significant survival following craniospinal irradiation for chemotherapy refractory disease presented. Further study on the use of craniospinal irradiation to treat leptomeningeal metastasis is recommended.

## 1 INTRODUCTION

Leptomeningeal metastasis (LM) is a relatively uncommon development in solid tumors, however, when it does occur, its consequences are associated with significant morbidity and mortality.^1^ LM is defined as tumor cell invasion of the pia, arachnoid mater, subarachnoid space or cerebrospinal fluid.^2^ The incidence of LM from solid tumors is approximately 10%,^3^ with breast cancer contributing to the majority of all LM cases (12–64% of cases) followed by lung cancer and melanoma.^4^, ^5^, ^6^ The incidence appears to be increasing as improvement of systemic and local therapies increase patient overall survival.^7^, ^8^ Despite improvements in therapy, morbidity from LM disease itself and the toxicities from treatment, remain significant. Symptoms of the disease are variable and may include cranial nerve palsies, encephalopathy, seizures, radiculopathies, incontinence or motor deficiencies.^9^ The median survival for patients with LM is abysmal, ranging between 4 and 15 weeks with only 15% of patients being alive at 1 year.^10^, ^11^, ^12^

Robust treatment guidelines for LM do not exist because of poor prognosis, relatively low numbers and a lack of large randomized clinical data in the literature.^13^, ^14^ Radiation therapy has been shown to improve quality of life, although, its effects are often short lived.^15^, ^16^, ^17^, ^18^

Although palliative radiation is often indicated for patients with LM, the role of craniospinal irradiation (CSI) is less defined. Most commonly, the recommendation in the literature is to avoid CSI for the treatment of LM except for select cases.^19^, ^20^ There is sparse data to support these recommendations, especially given the tremendous advancements in radiation therapy technology and techniques.

We present a case of a woman suffering from LM with excellent response to CSI and survival well above the median despite minimal response to intrathecal therapy.

## 2 CASE REPORT

Mrs. M. is a 50‐year‐old woman with a history of metastatic breast cancer who presented with a 2‐week history of headaches, nausea, and vomiting. History and physical exam revealed no photophobia or focal neurologic deficits, however, generalized lower extremity weakness reduced her ability to ambulate significantly below baseline. Considering her history of metastatic breast cancer, a magnetic resonance imaging of the brain was ordered which showed a stable or slightly reduced size of a previously treated lesion in the left insular cortex. Given the ongoing symptoms, lumbar puncture with CSF cytology was performed, which confirmed metastatic carcinoma consistent with breast primary, establishing a diagnosis of LM.

Mrs. M had been diagnosed 12 years prior with a T2 N0 multicentric lobular breast carcinoma at the age of 38. She was treated with lumpectomy, axillary lymph node dissection followed by completion mastectomy, adjuvant chemotherapy and chest wall radiation. This was followed by 5 years of tamoxifen therapy. Ten and a half years after her initial diagnosis at the age of 48 years, her cancer recurred in her brain and bones. She received stereotactic radiosurgery to a solitary, gradually growing lesion in her left insular cortex. At the same time, she was found to have lesions in her thoracic spine and right scapula. She received a single fraction of radiation to her scapula and was restarted on tamoxifen with excellent response in her thoracic spine. She went just over 1 year without recurrence.

Upon her diagnosis of LM, Mrs. M received intrathecal methotrexate and cytarabine with no resolution of her symptoms and persistently positive CSF cytology. She was then switched to six cycles of intrathecal liposomal cytarabine along with oral capecitabine. Despite several cycles of intrathecal and systemic chemotherapy treatments there was no clearance of her CSF cytology and she had only partial resolution of her headaches and nausea. There was no treatable solid tumor identified on CT or MRI imaging of her brain to account for her symptoms.

**FIGURE 1:**
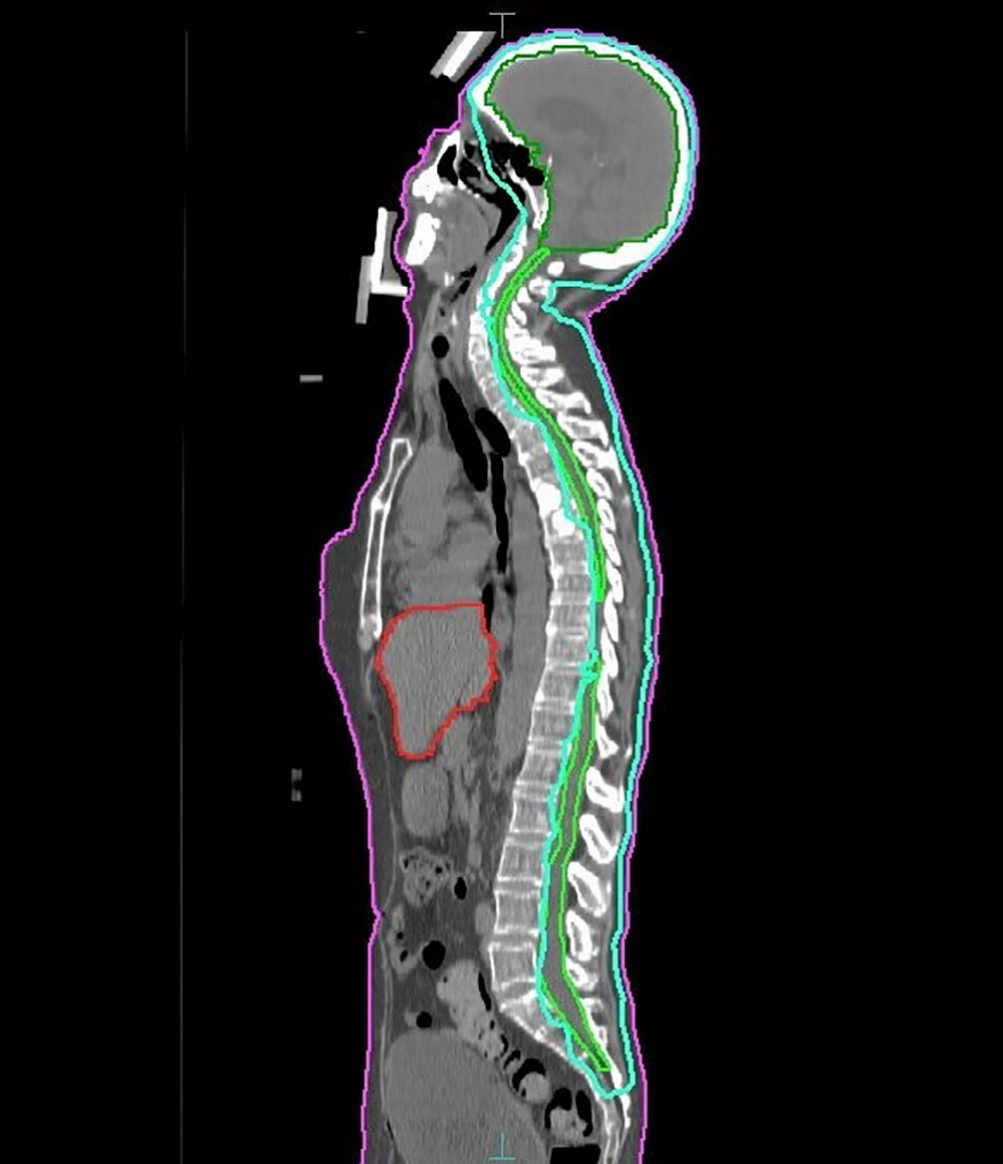
Craniospinal irradiation treatment plan for Mrs. M. showing the 95% isodose line (aqua blue line) covering the whole brain, meninges and the anterior spinal canal

Mrs. M's case was discussed at multidisciplinary tumor boards. Given the persistent positivity on CSF cytology it was decided she would receive palliative CSI to a dose of 36Gy in 20 fractions over 4 weeks time prescribed to cover the anterior spinal canal with 95% of the prescription dose (Figure 1). She had significant toxicities during this treatment, including sore throat, odynophagia, and significant fatigue. Of significance, despite being on a relatively modest dose of dexamethasone, she also developed severe proximal muscle weakness requiring an early and rapid tapering of her steroid. This increased her fatigue, nausea and general malaise on treatment.

Following her radiation, her acute side effects rapidly resolved over 4–6 weeks. Her proximal muscle weakness was slower to respond, nonetheless she was ambulating short distances with a walker at 1 month of follow‐up. After 3 months, her strength had almost returned to baseline and she was walking slowly, without assistance. She remained on tamoxifen and received 1 year of oral capecitabine.

**FIGURE 2:**
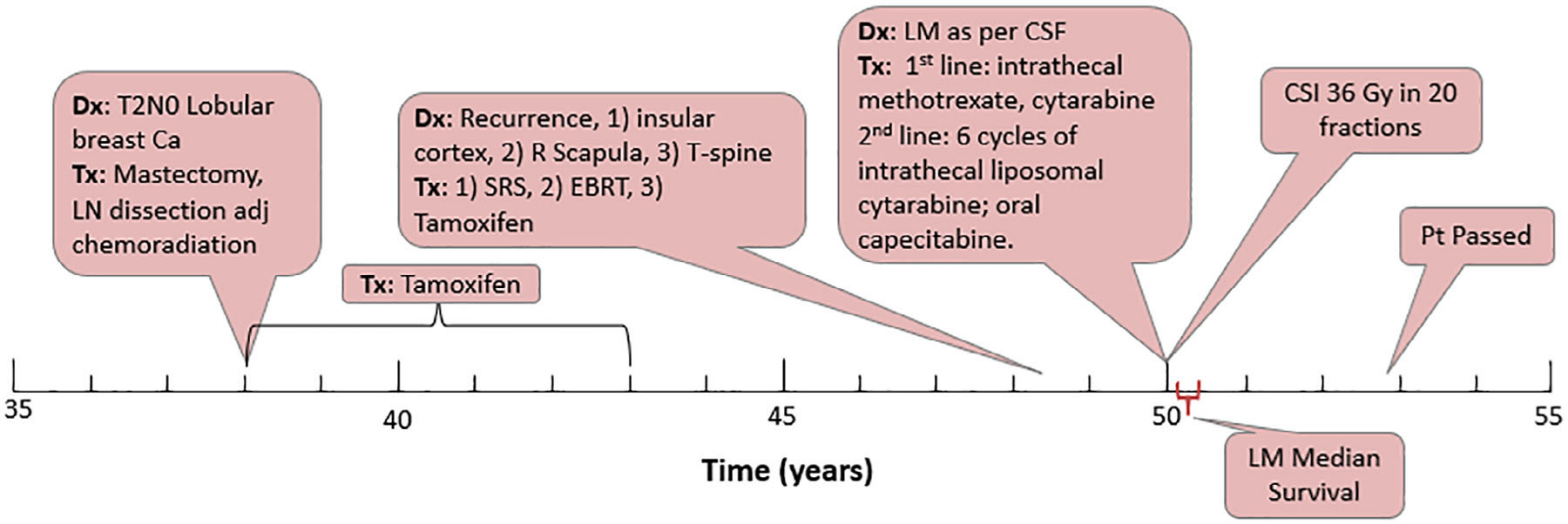
Approximate timeline of Mrs. M's Breast Cancer history, from diagnosis, throughout her treatment and disease progression. Leptomeningeal metastases median survival is indicated for comparison. Ca, cancer; LN, lymph node; adj, adjuvant; T‐spine, thoracic spine; SRS, stereotactic radiosurgery; EBRT, external beam

She did very well for 2 years following her radiation treatment, at which point she developed rapid vision loss and was found to have hydrocephalus requiring a ventriculoperitoneal shunt. Vision and general strength did not fully recover following this and she declined despite the procedure. The cause of her hydrocephalus was never clear but was not aggressively investigated due to the rapid deterioration of the patient's health at this point. Ultimately, she died from her disease 2 years and 11 months following her initial presentation with LM and 2½ years following her CSI (Figure 2).

## 3 DISCUSSION

Chemotherapy, typically intravenous or intrathecally administered, is the first‐line treatment modality for LM disease. However, chemotherapeutic options are limited in LM disease because of the poor blood‐brain barrier penetration of most intravenously or orally administered agents.^21^, ^22^, ^23^ The three main agents used intrathecally for LM treatment are methotrexate, cytarabine and thiotepa.^24^, ^25^, ^26^ In this case, we have a patient who has disease refractory to standard chemotherapy agents for LM leaving radiation therapy as a salvage palliative therapy.

Overall, studies support the inclusion of radiation therapy for the treatment of LM^7^ although there is not a lot of evidence on the use of CSI. CSI is a method that is used sparingly in the treatment of patients with LM because of its technically complicated set‐up, the overall poor prognosis with LM and the association of CSI with pronounced toxicities such as myelosuppression, mucositis, nausea and dysphagia. Current literature frequently highlights the limited indications of CSI in LM due to its many toxicities and its small documented survival benefit. A review by Le Rhun et al suggested that “focal radiotherapy is commonly indicated for macroscopic disease,” while the role of whole brain radiation therapy “is decreasing,” and CSI “is rarely an option for LM from solid cancers” because of the risk of side effects.^8^ The National Comprehensive Cancer Network (NCCN) guidelines for LM suggest giving stereotactic radiation, surgery, involved field or whole brain radiation therapy to bulky disease, neurologically symptomatic or painful sites. It states, CSI should only otherwise be considered in “highly select patients [with radiosensitive disease] (e.g., leukemia, lymphoma).”^20^

Four retrospective reports in the literature examine series of patients with LM treated with CSI.^27^, ^28^, ^29^, ^30^ All studies concluded that CSI is feasible and effective in select patients. They suggested overall survival with CSI in their cohorts to be similar to that of other patient cohorts treated without CSI but with palliation of symptoms achieved. Combined modality therapy seemed superior to irradiation alone. El Shafie et al showed on multivariate analysis that age, performance status and neurologic response to therapy predicted longer overall survival. In this study, 3 of 25 patients survived past 1 year and one patient survived for over 2 years.^29^ In a more recent study by Devecka et al, it was demonstrated that patients had a better overall survival (median of 7.3 months compared to 1.5 months) with both a Karnofsky performance scale index of >70% and the absence of extra‐central nervous system disease.^27^ This suggests there may be a LM subpopulation that benefits from CSI.

Recently, Yang et al reported a Phase I prospective trial using hypofractionated proton CSI for the treatment of LM disease. Twenty‐four patients were enrolled with the median progression‐free survival being 7 months and overall survival being 8 months. Four patients were alive and free from central nervous system progression for more than 12 months. The authors reported that adverse effects from proton CSI are far less prevalent than for what is reported for traditional photon CSI.^31^ While this study uses a different radiation modality than was used in our patient, it further suggests that in select patients CSI can result in prolonger survival.

We report here a case of a patient who received craniospinal radiation for LM and survived 2 years and 11 months, a time well above the median stated in the literature, with a reasonable quality of life. This was despite no response to intrathecal chemotherapy. To our knowledge, this is the first case reported with long‐term survival following treatment of LM with salvage CSI. This was a patient that was relatively young, had minor neurological symptoms from her disease and was refractory to chemotherapy. This may represent a sub‐population with LM that benefits from CSI. Caution in patient selection is still required, however, due to the significant acute side effects of CSI. There are remarkably few recent studies in the literature examining the expansion of the therapeutic role of CSI for LM, highlighting the need for further inquiry.

## 4 CONCLUSION

With improving radiation and systemic therapies, newer data is required to revisit the LM treatment paradigm. Despite a declining use and lack of mention in guidelines and review, the use of CSI for LM can have good survival and quality of life outcomes for certain patients despite potentially severe acute toxicities on treatment. CSI should be considered in LM from solid tumors, particularly in younger patients with good performance status and potentially in patients who have failed intra‐thecal chemotherapy. More research is required to determine the subpopulations of patients who might benefit most from CSI.

## CONFLICT OF INTEREST

The authors declare no conflict of interest.

## AUTHOR'S CONTRIBUTIONS

*Methodology, Writing—Original Draft, Writing—Review and Editing*, D.T.; *Writing—Review and Editing*, D.V.; *Conceptualization, Methodology, Writing—Review and Editing, Supervision, Project Administration*, K.R.

## ETHICAL STATEMENT

This report was waived for review by the Thunder Bay Regional Health Sciences Centre Ethics Review Board. Formal decision on August 5, 2021. Written consent was obtained from next of kin to publish the above information.

## Data Availability

Data sharing is not applicable to this article as no new data were created or analyzed in this study.
